# Optical-Tactile Sensor for Lump Detection Using Pneumatic Control

**DOI:** 10.3389/frobt.2021.672315

**Published:** 2021-07-01

**Authors:** Jonathan Bewley, George P. Jenkinson, Antonia Tzemanaki

**Affiliations:** ^1^ Department of Mechanical Engineering, Faculty of Engineering, University of Bristol, Bristol, United Kingdom; ^2^ Bristol Robotics Laboratory, University of Bristol, Bristol, United Kingdom

**Keywords:** tactile sensing, soft sensors, pneumatic actuation, variable stiffness, medical diagnosis, medical robotics

## Abstract

Soft tactile sensors are an attractive solution when robotic systems must interact with delicate objects in unstructured and obscured environments, such as most medical robotics applications. The soft nature of such a system increases both comfort and safety, while the addition of simultaneous soft active actuation provides additional features and can also improve the sensing range. This paper presents the development of a compact soft tactile sensor which is able to measure the profile of objects and, through an integrated pneumatic system, actuate and change the effective stiffness of its tactile contact surface. We report experimental results which demonstrate the sensor’s ability to detect lumps on the surface of objects or embedded within a silicone matrix. These results show the potential of this approach as a versatile method of tactile sensing with potential application in medical diagnosis.

## 1 Introduction

Increasingly, there is demand for robotic systems to operate within variable unstructured domains, such as in autonomous exploration or alongside humans (Zou et al., 2017). This requires continually gathering detailed information about the local environment to facilitate executing the task in a safe manner. Tactile sensing at the external interface of robotic end-effectors enables a direct feedback loop to modulate applied force during interactions (Zou et al., 2017). The adaptability of soft tactile sensors enables the contact surface to conform to the shape of objects and distribute any handling forces over a larger contact area (He et al., 2020a).

In addition to the importance of tactile information when interacting within unknown environments, physical touch becomes highly important when other sensory information is unavailable (Tiwana et al., 2012). Despite research interest, commercial application of soft tactile sensors has been extremely low (Tiwana et al., 2012; Zou et al., 2017). Zou et al. (2017) highlight the high cost, low modularity and the advancements in micro manufacturing and software processing necessary to implement research sensors practically as key challenges to their adoption. By tackling these problems and working toward more generally applicable soft tactile sensor technology, we hope to broaden the range of applications for which robotic systems can be utilized and enable tasks, previously only possible with partial or total human intervention, to be completed autonomously.

A wide range of tactile sensing technologies has been developed with successful designs coming from both close biomimicry as well as deviating from nature to incorporate additional capabilities (Zou et al., 2017; Chi et al., 2018). One such deviation proposed in literature comprises adjusting the effective stiffness of the soft tactile membrane via pneumatic actuation in order to allow for the sensitivity and measurement range of the sensor to be adjusted, leading to a more generically applicable sensor (He et al., 2020a; Jenkinson et al., 2020; Zhang et al., 2021). Previous studies exploring pneumatic actuation within soft tactile sensors have applied this to reactive grasping (McInroe et al., 2018), shape identification (Huang et al., 2019; Xiang et al., 2019), estimating tissue elastic modulus (Gubenko et al., 2017) and an explorative capsule capable of self-locomotion (Hinitt et al., 2015). Variable effective stiffness tactile sensors have also been applied to emulate nodules inside phantom organs to assist with medical diagnosis training (He et al., 2020b).

A key challenge when incorporating sensors within soft robotics systems is to not alter the overall compliance of the robot’s external interfaces (Shintake et al., 2018). Optics-based tactile sensing is common across soft actuated sensors, as all electrical components can be physically separated from the tactile membrane (Shimonomura, 2019). Among the pneumatically actuated tactile sensors referenced above, only He et al. (2020a) and He et al. (2020b) use pneumatic variations as the primary form of sensing; all other studies chose to implement optics-based tactile sensing. These studies have focused on detecting surface or bulk features of the stimulus that they are sensing, where heterogeneity in the depth of the stimulus remains under-exploited as a tactile cue.

In this work, we explore the potential of pneumatically actuated soft tactile sensors though the development and experimental characterization of a novel sensor. We investigate the usefulness of varying the effective stiffness of the sensor’s tactile membrane when identifying nodules embedded in a soft medium, coarsely imitating the manual palpation tasks required for clinical breast examinations (CBEs).

CBEs serve a vital role in monitoring breast health and reducing the number of patient referrals to the more costly procedures of MRI or X-ray mammography, where many users report an unpleasant and painful experience (Bickley et al., 2013; Whelehan et al., 2017). These procedures are the current gold standard for medical diagnosis but are uncomfortable and their relatively high rate of malignant diagnosis of benign tissue (false positives), especially in younger women, can lead to patient anxiety and further unnecessary invasive treatment (Bickley et al., 2013). If data-led CBEs were to become a frequent and automated process, monitoring of breast health over time could be streamlined and anomalies identified through tactile sensing and machine learning. However, an automated breast examination system would require tactile sensitivity at least comparable to that of a trained healthcare professional performing CBE in order to achieve this (Tiwana et al., 2012).

**FIGURE 1:**
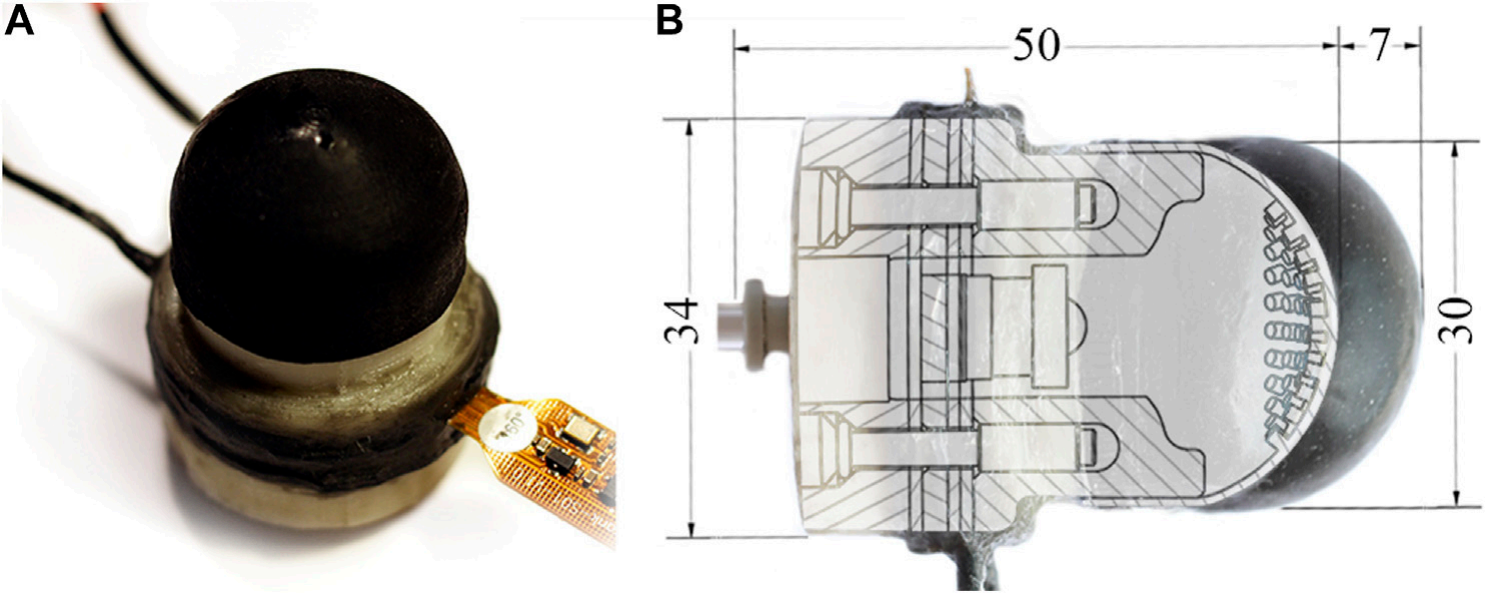
**(A)** The soft actuating tactile sensor. **(B)** A sectioned view demonstrating the sensor’s internal structure as well as its potential for active palpation. Pressurizing the sensor achieves linear actuation (max of 7 mm) through expanding the soft tactile membrane, and thus additionally modifies its effective stiffness.

Figure 1 depicts the proposed sensor along with its internal structure. The soft external membrane of the sensor deforms in response to tactile stimuli, as the internal pressure and motion of embedded tracking pins are monitored and analyzed. The tactile information is represented with the use of Voronoi diagrams as per the work of Cramphorn et al. (2018). We show that this approach is capable of resolving a broad range of tactile stimuli. We validate the sensor’s ability to distinguish surface lump of varying size and then characterize the sensor’s performance at detecting lumps embedded within a silicone matrix as the effective stiffness of the tactile membrane is adjusted.

The following sections focus on the design and fabrication of the compact, low-cost soft tactile sensor incorporating pneumatic actuation (Sections 2.1 and 2.2) and its characterization (Section 2.3). Section 3.1 presents a machine learning approach for identifying the presence of lumps utilizing features derived from Voronoi tessellation. The results demonstrate that the sensitivity of the sensor to identify lumps in a CBE-inspired task can be improved by pneumatically tuning the effective stiffness of the tactile membrane (Section 3.2). Section 4 discusses the results and makes recommendations for future work.

## 2 Materials and Methods

**FIGURE 2:**
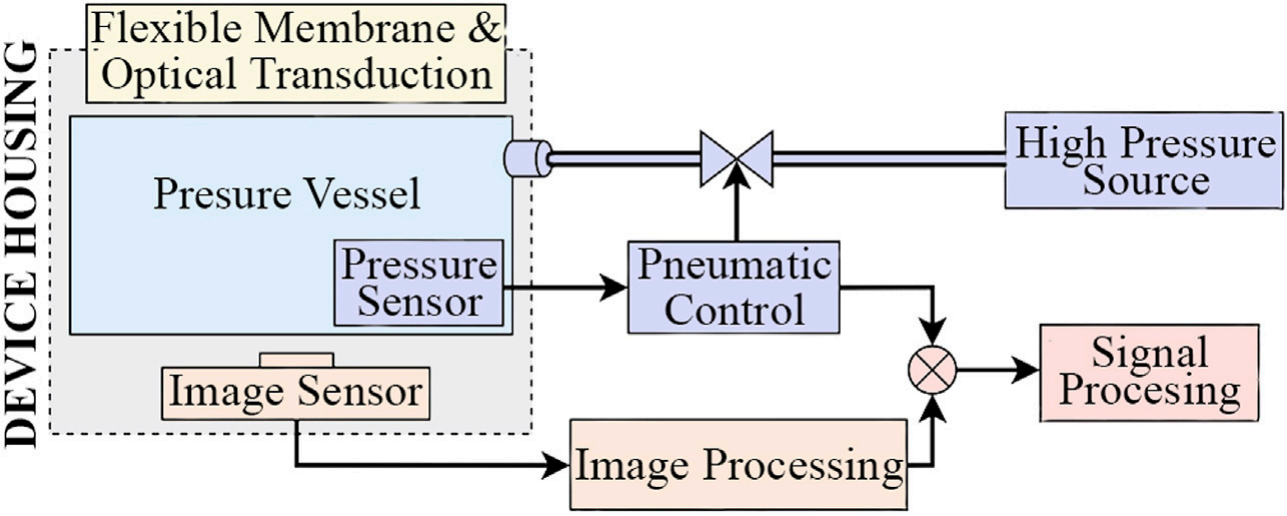
High level system diagram for an optical based tactile sensor incorporating pneumatic actuation.

Incorporating pneumatic actuation within an optical based tactile sensor poses restrictions on the form the device may take: the sensor’s cavity needs to be pressurized and the image sensor must have unobstructed view to the tactile membrane. McInroe et al. (2018) tackled these same restrictions in the design of their sensor. Figure 2 shows a high level system diagram of such a system.

### 2.1 Design and Fabrication

#### 2.1.1 Tactile Membrane

**FIGURE 3:**
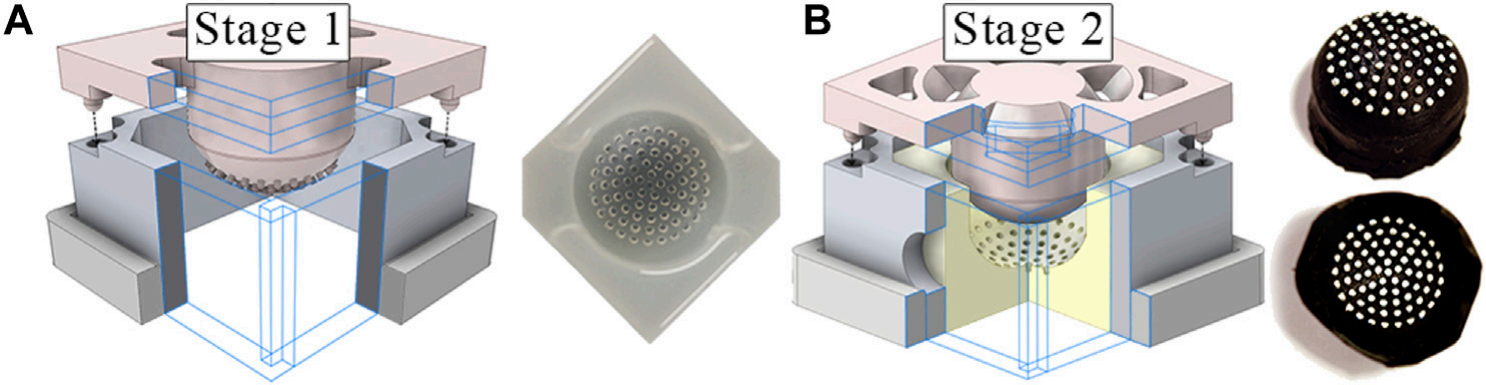
**(A)** Two step molding process to form the tactile membrane using 3D printed molds. The clear negative mold, formed in the first stage, is used in the second stage to form the inverted tactile membrane. **(B)** Lastly, the membrane is flipped ‘inside-out’ and the pin tips are painted white.

The tactile membrane serves to conform to surfaces the sensor comes into contact with so that the tactile stimuli can be converted to a form which the internal sensors can detect. It is manufactured using a silicone molding methodology resembling that depicted by Winstone et al. (2012) and common across the early sensors within the TacTip sensor family (Ward-Cherrier et al., 2018). Figure 3 depicts the two-stage molding process.

The membrane features 72 pins (of the same material as the membrane), 1.2mm long and of 1.25mm diameter, distributed in radially offset concentric rings (Figure 3B). This arrangement allows for simple parameterization of the silicone molding CAD (Computer-Aided Design) models while maintaining an approximately uniform marker distribution. With the membrane inverted, the painted pin tips are spaced 1.75mm apart with a maximum deviation between marker centers of ±0.12mm. The tactile membrane is a flattened dome, 10mm high and with a diameter of 30mm (see Figure 1). The variable internal pressure (implemented by pneumatic actuation, see Section 2.1.3) allows for the curvature of the membrane to be altered during operation. An initial profile flatter than a hemisphere was chosen to enable manufacture with low cost FDM (Fused Deposition Modeling) 3D printers and facilitate removal of the soft tactile membrane and its ‘inside-out’ pins from the silicone molds (see Figure 3).

The tactile membrane is moulded using DragonSkin 30 silicone (Smooth-On, Macungie, PA). We carried out Finite Element Analysis (FEA) to determine the suitability of DragonSkin 30 and subsequently tune the membrane thickness and operating pressure for desired behavior. We used Abaqus CAE to carry out nonlinear analysis by approximating the dome geometry as a plane stress shell with quadrilateral elements, fitting a polynomial to experimental stress-strain data and using a third-order Ogden model of hyperelasticity. A membrane thickness of 1mm proved sufficient to achieve a 100% volume expansion across a suitable working pressure range while remaining a safety factor of two below the maximum tensile stress of the material.

**FIGURE 4:**
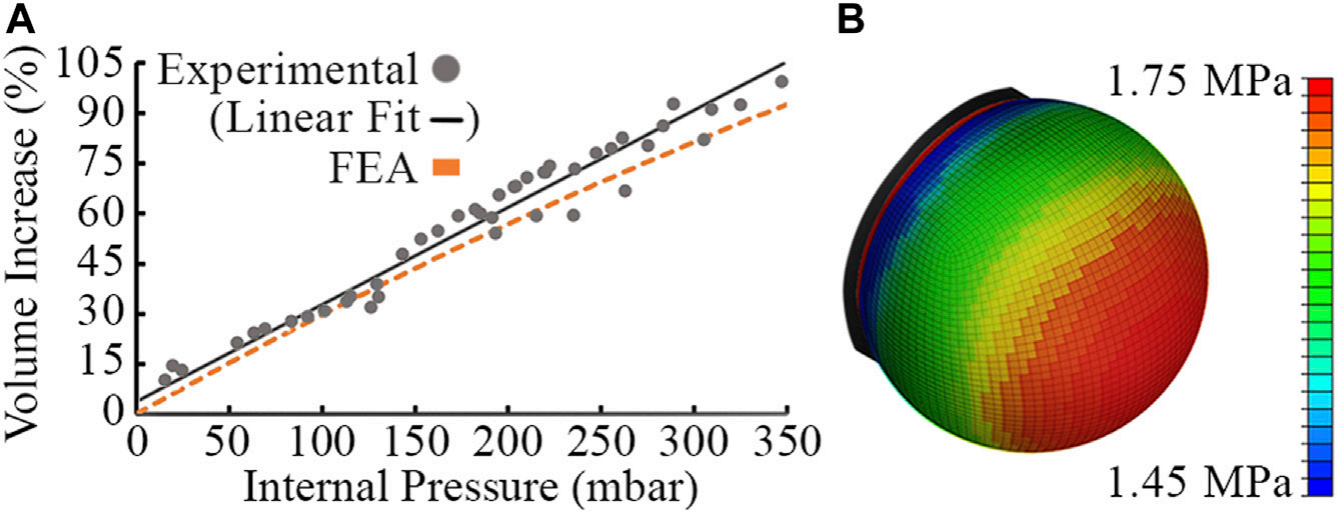
Experimental data points, linear fit of experimental data and FEA model using Abaqus for a 1mm thick DragonSkin 30 membrane. **(A)** Volumetric expansion against pressure predicted by the FEA model and found experimentally from the manufactured membrane. **(B)** FEA deformed mesh and stress distribution at 100% inflation.

The FEA model exhibited a cubic relationship between the internal pressure of the cavity and the percentage volume increase of the membrane for pressures between 0−1000mbar. Figure 4A compares the FEA predictions to experimental data specifically for internal pressure between 0−350mbar. Across this limited range, the relationship appears approximately linear and close agreement can be seen between the FEA model and experimental data. However, no conclusions can be drawn about experimental behavior at higher pressures. The experimental data were captured by filming the sensor expanding, tracking the pixels making up the side projection of the tactile membrane and assuming axisymmetric behavior to calculate the volume of revolution. This experiment was repeated three times and averaged.

#### 2.1.2 Embedded Sensing

A Raspberry Pi Zero camera module with a wide angle variable focus lens is used to capture optical changes of the tactile membrane, the underside of which is illuminated by a white LED. A 2bar Honeywell ABP digital pressure sensor is positioned in the pneumatic loop outside of the housing to record pressure fluctuations. The wide field of view, $160^{\circ }$, and zero minimum focal distance of the camera module allows the void space behind the tactile membrane to be minimized, while a frame rate of 90 fps enables marker motion to be tracked smoothly between frames. The compact sensor housing along with short lengths of narrow diameter tubing minimize the closed loop air volume such that pressure readings are reactive to external stimuli without delay.

#### 2.1.3 Actuation and Control

The internal pressure of the sensor may be altered to actuate the membrane and alter its geometry while emulating different stiffnesses. An Arduino serves to control the pressurization of the sensor cavity and then isolate it such that pressure fluctuations due to external stimuli on the tactile membrane can be detected. The pneumatic system is actuated by manually depressing a syringe while the Arduino monitors the internal pressure and closes the solenoid valve once the desired cavity pressure has been reached.

A Raspberry Pi Zero coordinates the system and is used to control the camera module and communicate with the Arduino to collect pressure readings and set the trigger pressure at which the solenoid valve is closed.

#### 2.1.4 Sensor Housing

**FIGURE 5:**
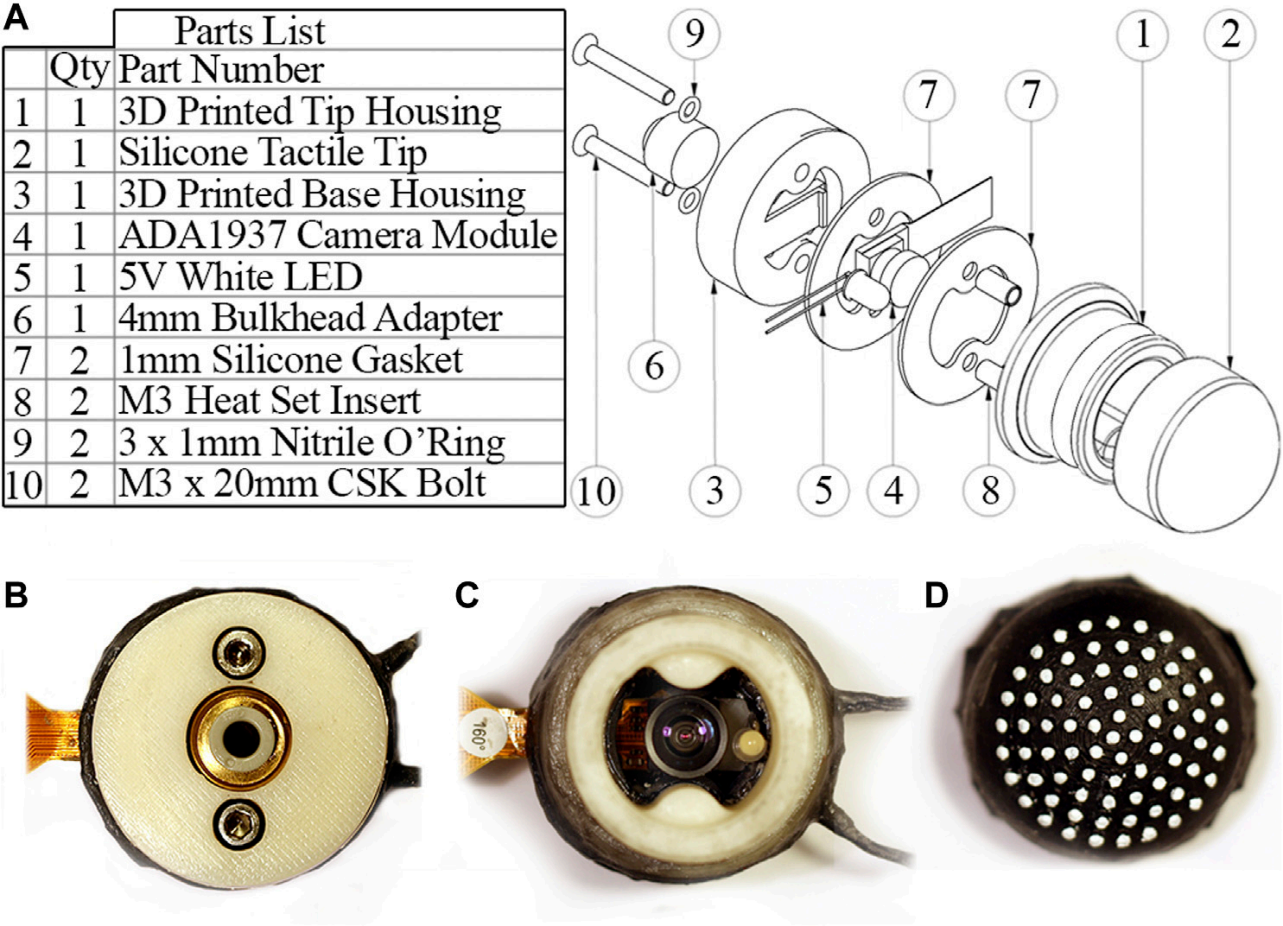
**(A)** Part list and exploded view of the sensor housing assembly. **(B)** Sensor underside with bulkhead adapter for connecting pneumatic tubing. **(C)** Sensor internals with tactile membrane removed. **(D)** Inside of tactile membrane.

Figure 5 shows an exploded view of the housing assembly along with images of the assembled sensor. The housing is 3D printed and split into a base section to mount the bulkhead adapter and camera module, and a tip section to mount the tactile membrane. An air-tight seal is achieved through threading the bulkhead adapter with PTFE tape and linking the PLA components with silicone gaskets under a high clamping force. A latex band attaches the tactile membrane to the removable tip section of the housing along a shallow groove. This makes the tactile membrane a modular component which can be removed and replaced due to wear or sanitary constraints, e.g. in a medical setting.

### 2.2 Tactile Signal Processing

**FIGURE 6:**
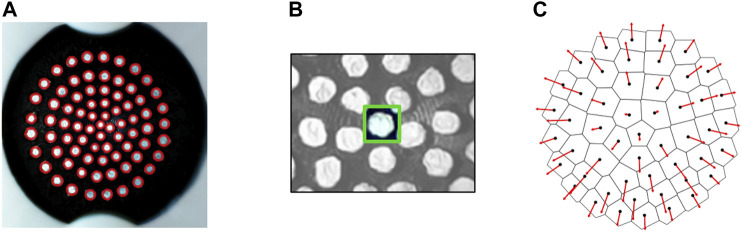
**(A)** Marker detection with SimpleBlobDetector algorithm tuned to identify white circular blobs against a black background. **(B)** Individual trackers generated for groups of pixels in a bounding box around each marker and tracked across frames using the TrackerKCF algorithm. **(C)** Voronoi diagram and marker displacement vectors calculated relative to the undeformed state.

Figure 6 shows the stages of image processing carried out on each frame using functions from the OpenCV library (Bradski, 2000). The center point of each marker is used to construct a Voronoi diagram (Figure 6C) using the SciPy library spatial. Voronoi function (Virtanen et al., 2020) and calculate displacement vectors from the initial non-deformed state. Calculating Voronoi diagrams from marker based tactile data, as proposed by Cramphorn et al. (2018), enables the divergence of markers away from each other to be quantified and visualized as the area change of the Voronoi region associated with each point.

The pressure signal is filtered to remove noise, synced with the image sequence and averaged over each frame interval to allocate each frame a single pressure sample point. Lastly, regions within the Voronoi diagram are uniquely numbered based on the angle and radius of each tracked point from the point cloud centroid. This allows regions to be indexed consistently across frames and monitored over time.

### 2.3 Sensor Characterization

**FIGURE 7:**
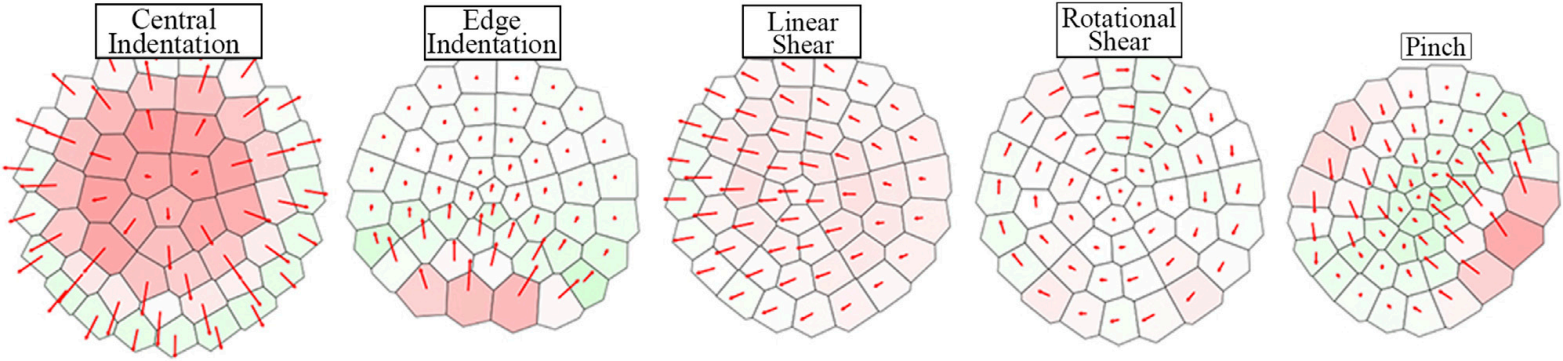
Tactile Voronoi diagram response corresponding to a range of stimuli. Voronoi regions are colored by increasingly dark shades of red or green to display area expansion or contraction respectively. The red arrows from the centroid of each region show the vector displacement of regions from their non-deformed positions.

Figure 7 qualitatively depicts the sensor’s response to a range of stimuli. The color of each region corresponds to its percentage area change (expansion/contraction). The responses are distinct and display the sensor’s ability to resolve tactile information. The area change and vector displacement of the Voronoi regions encode different tactile information and hence provide a more complete representation of the stimuli when combined.

**FIGURE 8:**
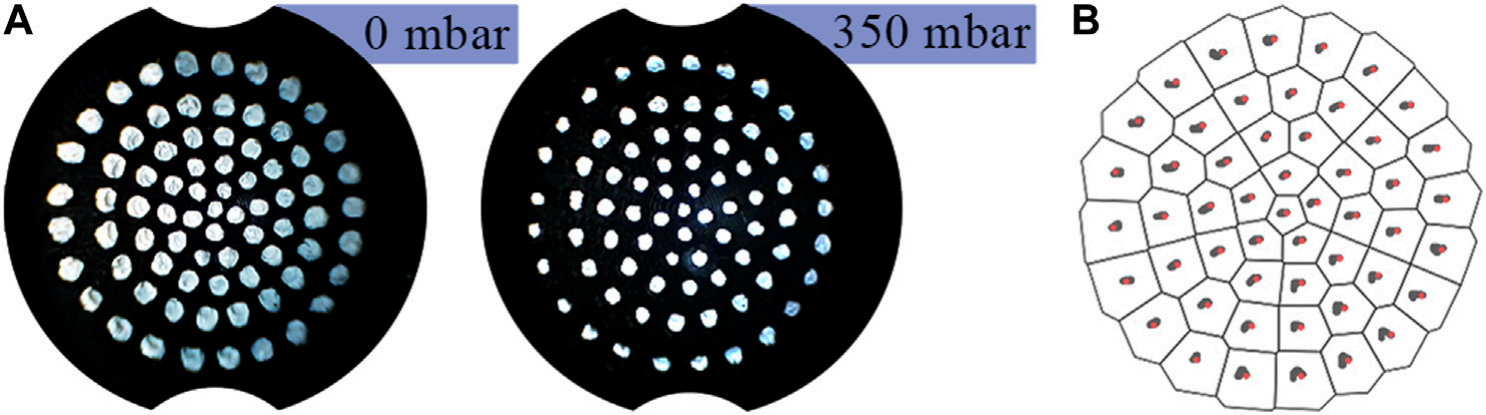
**(A)** Marker distribution at 0bar and 350mbar internal pressure. **(B)** Marker centers superimposed on top of each other as the internal pressure is increased from 0mbar to 350mbar.

Figure 8 shows the effect of pressure change on the marker distribution. Despite the membrane geometry changing significantly, Figure 8B demonstrates how the observed distribution of markers is near invariant of pressure. This invariance means that internal pressure need not be accounted for during image processing.

**FIGURE 9:**
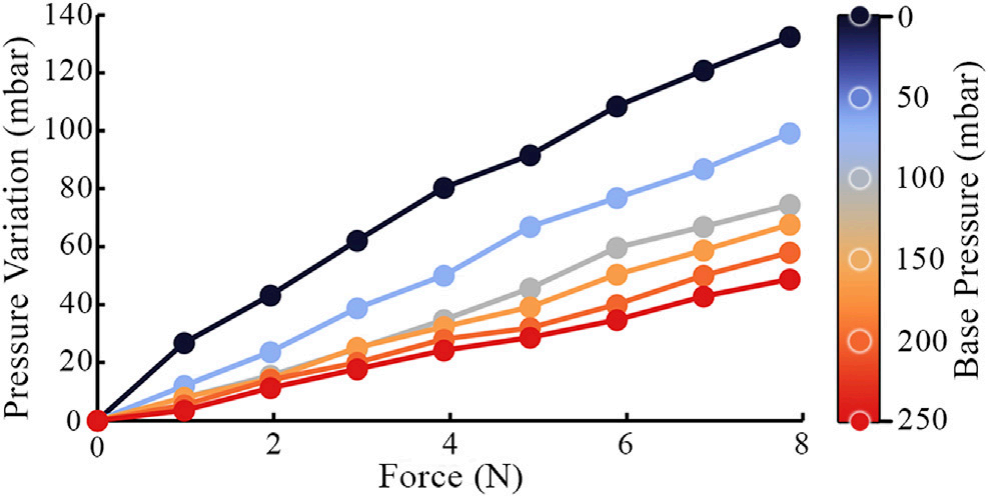
Internal pressure variation due an external force deforming the tactile membrane. Each data series is recorded at a different baseline internal sensor pressure.

The tactile membrane can be inflated and deflated by the pneumatic control system described in Section 2.1.3. Additional indentation into the membrane (due to contact with a surface/lump) will further increase this pressure. The relation between internal pressure and an external force applied through a flat plate normal to the membrane is shown in Figure 9 for different baseline pressures of the sensor’s cavity. Increased baseline cavity pressure results in increased effective stiffness of the membrane, i.e., how much it will resist deformation in response to a given force. In addition to the baseline cavity pressure, applying further force to the sensor increases the internal pressure, and therefore the effective stiffness. Over the investigated force range of the sensor, the relationship between the applied normal force and the resulting pressure variation appears approximately linear. However, over a larger force range, the curve for each baseline pressure would visibly flatten as the membrane stiffens (Jenkinson et al., 2020). By fitting a curve to this relationship, the pressure sensor could be used to approximate a normal force applied to the membrane, given that the baseline internal pressure is known.

## 3 Lump Detection

CBEs aim to identify anomalous hard lumps within a search domain of breast tissue. In the next two sections, we characterize the relationship between the presence of an anomalous lump and the area change and vector displacement of the 51 regions of the Voronoi diagram, in order to assess the performance of the presented sensor for lump detection. First, we consider the problem of detecting surface lumps, followed by considering embedded lumps, in which case this mapping becomes increasingly non-linear, due to the complex dynamics of elastic deformation.

### 3.1 Surface Lumps

**FIGURE 10:**
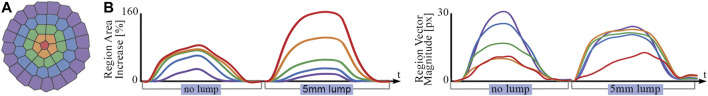
**(A)** Voronoi regions colored by radial distance from centroid. **(B)** Ring-averaged area and vector displacement magnitude response to a flat plate (no lump) in comparison to a 5mm surface lump. This example shows data taken at a baseline pressure of 50mbar and with a normal indentation force of 3.5N into the surface. When a 5 mm lump is present, the central rings’ response changes significantly compared to the outer rings’.

Averaging Voronoi regions in rings maintains only a single spatial dimension in the radial direction, thus making the assumption that the response is axisymmetric. This assumption is uniquely suitable for the application of detecting anomalous lumps through central indentation as the stimuli itself is approximately axisymmetric. Figure 10 shows how Voronoi regions can be grouped by their radial position and how this perspective illuminates clear differences in response when a surface lump is present. As the indentation is positioned centrally on the tactile membrane, Voronoi region area increase is predominately in the inner rings, whereas the vector displacement response is predominantly in the outer rings as the membrane spreads out toward to fixed boundary.

The response of the sensor was captured for five sizes of hemispherical surface lumps ranging in diameter from 1mm to 5mm using a modified Ultimaker 3D printer to serve as a Cartesian robotic arm. Results were recorded at four different internal pressures, ranging from 0mbar to 200mbar in even increments of 50mbar, to investigate how the effective stiffness of tactile membrane impacts performance. Each unique pressure and lump size combination was repeated twenty times. The tactile membrane was gradually indented into each lump sample up to a normal indentation force of 8N. A set of digital scales were used to calibrate the palpations to ensure a consistent indentation force.

**FIGURE 11:**
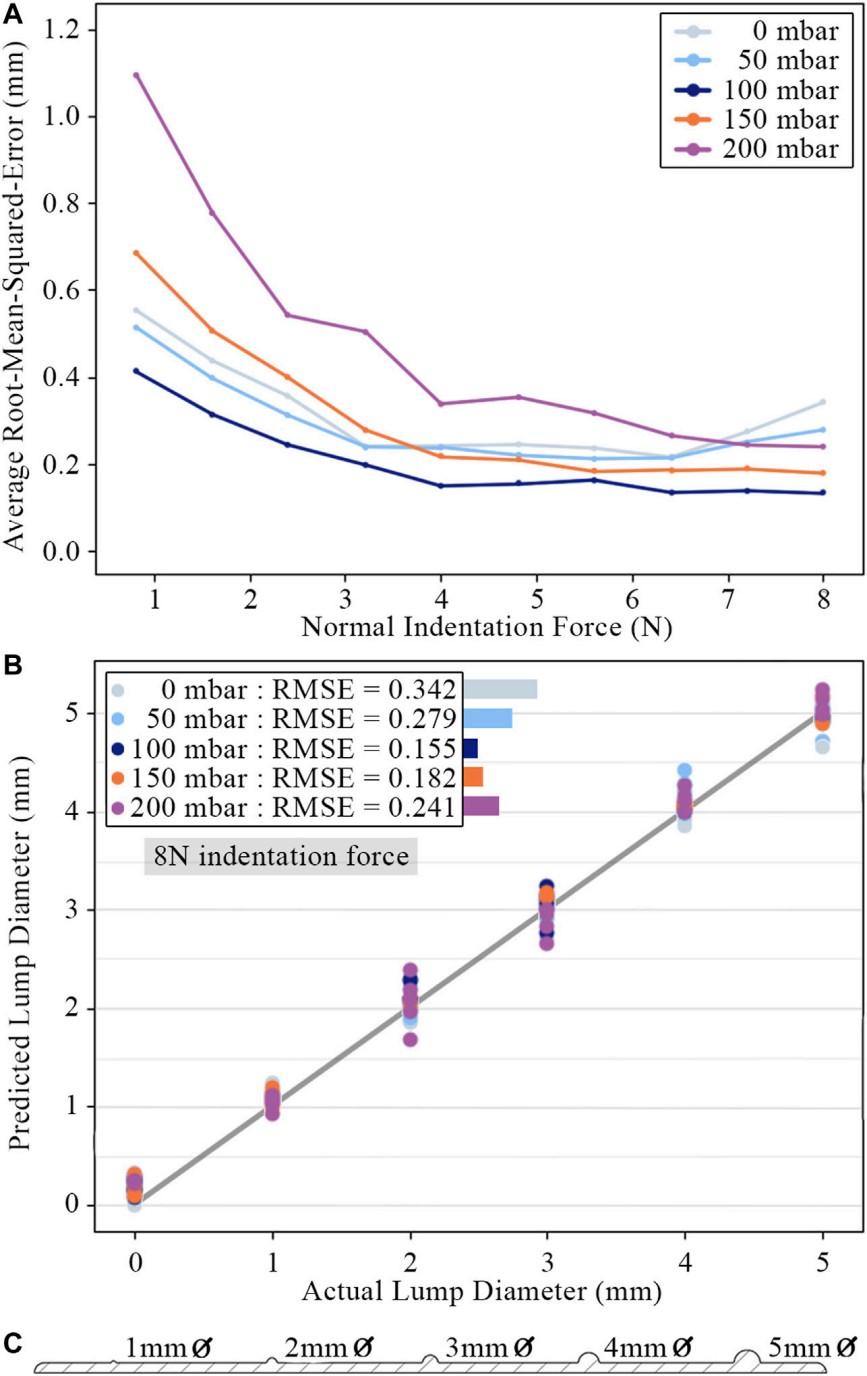
**(A)** Root-mean-square-error of lump size predictions for different internal pressures across a range of indentation force. Points plotted for each pressure and force are average values across all tests. **(B)** Predicted lump diameters for an indentation force of 8N. Each test is plotted as an individual point. **(C)** 3D printed lump test slab with hemispherical surface lumps varying in diameter from to 5mm.

In order to quantify the surface lump identification ability of the sensor, a linear regression model was trained using Voronoi diagram features to predict lump size. The scikit-learn library was used to implement and evaluate the regression model (Pedregosa et al., 2011). The model takes an input of ten features recorded at a single instance in time: the Voronoi region area change and vector displacements averaged around each of the five concentric rings. Region vector displacements are projected radially away from the marker centroid before averaging to isolate radial displacement from any lateral motion. Figure 11C shows the design cross-section of the 3D printed test slab used to gather data for the linear regression model. This data was partitioned into an 80/20 training/testing split and evaluated using 5-fold cross-validation. Figures 11A,B are the graphs of the model’s predictive performance on the testing data. Separate regression models were trained on Voronoi diagram frames at set intervals over the duration of each palpation so that performance could be compared for different indentation forces into the sample.

Accurate surface lump diameter predictions can be observed across the full range of internal pressures and lump sizes. The sensor can clearly distinguish lumps as small as 1mm from a flat surface. The range of predicted diameters for each lump are distinct, all within ±0.5mm of the actual value, and have an average standard deviation across all lump sizes and pressures of 0.13mm. The mean of the predicted diameters for each lump size were all correct within 0.08mm of the true values. During contact with a lump sample, the membrane must conform to the lump shape in order for the response to be distinct and identifiable. Higher internal pressures (and, therefore, higher membrane effective stiffnesses) display a clear trend of prediction accuracy increasing with indention force. While lower internal pressures are preferable at low indentation forces, the 0mbar and 50mbar pressurized cavities saw their prediction accuracy fall at forces above 6N. This suggests that with a high enough indentation force, the large scale deformation of the tactile membrane will ‘saturate’ the sensor and begin to mask the differences between stimuli. An internal pressure of 100mbar results in the lowest prediction error across all indentation forces and, hence, can be considered optimal for this specific set of stimuli.

### 3.2 Embedded Lumps

Detecting hard lumps embedded within tissue is a more challenging problem, as the surrounding tissue damps the response and adds additional non-linearity. Consistent with previous studies (Egorov and Sarvazyan, 2008; Gwilliam et al., 2010; Mojra et al., 2012) that compared the performance of commercial tactile sensors to human palpation, the response of the proposed sensor is investigated for vertical indentation into embedded lumps of different size and depth.

**FIGURE 12:**
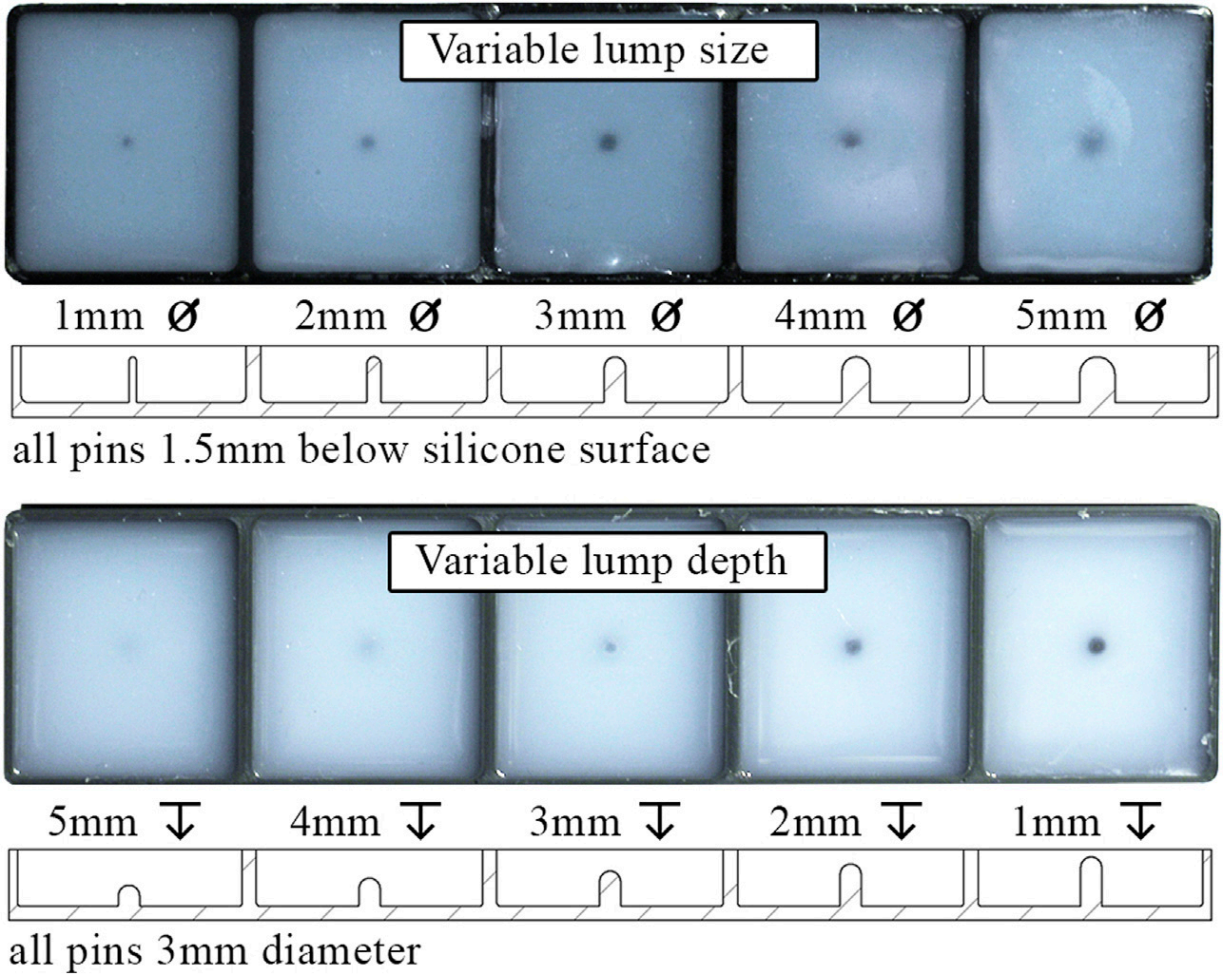
Top and sectioned views of test slabs with varying embedded lump size and depth.

Figure 12 shows the embedded lump test slabs, manufactured by encasing PLA lumps in DragonSkin 10 silicone. One test slab includes five lumps varying in diameter (1-5mm), all embedded at a depth of 1.5mm below the surface, while another test slab includes five lumps of 3mm in diameter, embedded in varying depths (1-5mm). Additionally, one test slab was manufactured with no embedded lumps to evaluate the likelihood of false positives. This experimental setup is similar to the silicone test slabs in Gwilliam et al. (2010) but approximately 10 times firmer than the medical breast phantom in Egorov and Sarvazyan (2008). Nevertheless, we consider these test slabs to be sufficient for an initial exploration of whether detecting hard lumps embedded in a softer matrix is possible.

Data were recorded across internal pressures ranging from 0mbar to 200mbar. Fifteen repetitions were recorded for each combination of test slab, internal pressure and indentation force. For the detection of embedded lumps, the same ten Voronoi diagram features were used to train a Bayesian classifier model. The approach outlined by VanderPlas (2016) was followed to implement a Gaussian kernel density estimator (KDE) with Bayesian classification using the scikit-learn library. By fitting multi-feature Gaussian KDE distributions to each lump class within the training data set, novel testing data can be assessed by calculating the likelihood that it belongs within each of the learnt distributions. The data set was partitioned and evaluated, as with the surface lump linear regression model, using a 80/20 training/testing split and 5-fold cross-validation.

**FIGURE 13:**
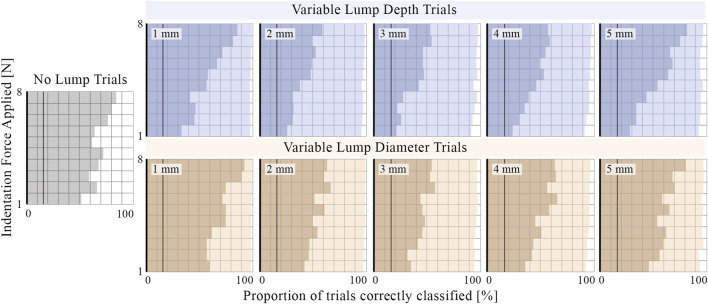
The proportion of trials where the presence of an embedded lump is correctly identified (light shading) and the proportion classified as lumps of the correct depth or diameter (darker shading). For comparison, vertical lines are plotted at 16.7% to indicate the accuracy of a dummy classifier implementing uniform random guessing across the six classes. Results are averaged across readings from all baseline internal pressures (0-200mbar) and shown across a range of indentation forces up to 8N. The presence of smaller diameter lumps embedded at shallower depths are more easily identified. The depth and diameter classification accuracy of identified lumps is low with light indentation force but improves as greater indentation force is applied.

Figure 13 shows the sensor’s ability to distinguish various embedded lumps from the surrounding silicone matrix and correctly classify lumps by their depth or diameter. Increased indentation force decreased the proportion of false positives in trials where there was no lump present (improving specificity) and increased the proportion of lumps whose presence was successfully identified (improving sensitivity) and correctly classified. The improvement in both identification and classification ability from increasing indentation force is greater for the variable depth trials, which demonstrates an increased need for use of higher force when deeper lumps are considered. The presence of lumps which are located closer to the surface and which have a smaller diameter is easier for the sensor to detect. The tactile signature from central indentation into a larger diameter lump is distributed over a larger area and, due to the choice of features and learning model, is more likely to be mistaken for the no-lump class. Lumps that are identified but incorrectly classified are most commonly mistaken for neighboring classes. This indicates that the model has successfully learnt the ordered relationship between the classes and provides an explanation for why the embedded lumps at either end of the investigated range appear to be correctly classified with greater accuracy.

**FIGURE 14:**
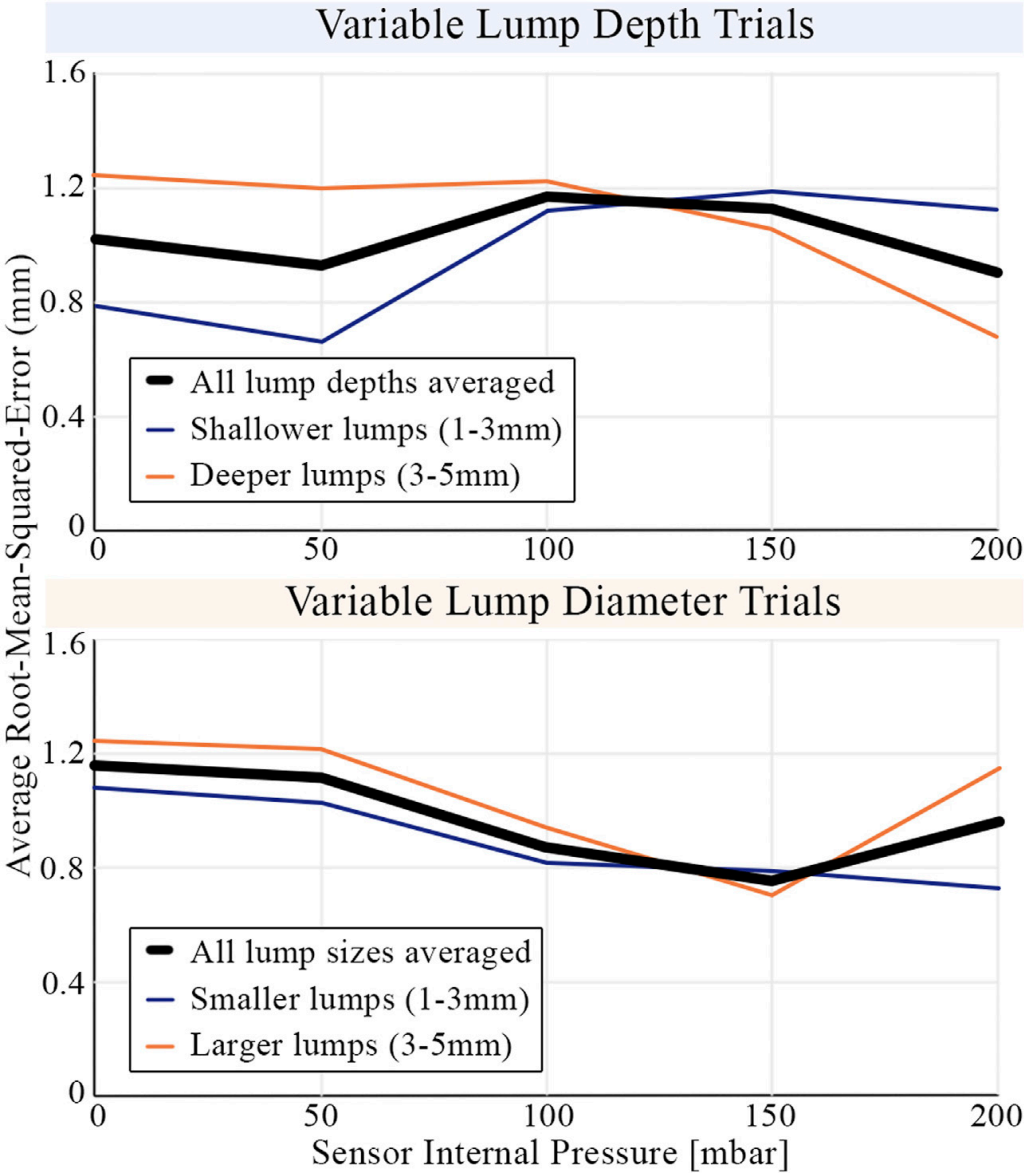
The average root-mean-squared-error in classifying embedded lumps by their size and depth. Results are shown averaged across all indentation forces (1.6N to 8N). Additional lines are plotted averaged across different subsets of embedded lump trials to highlight differences in behavior. The sensor’s accuracy in identifying the size and depth of embedded lumps varies as the internal pressure of the sensor, and therefore the effective stiffness of the tactile membranes, is adjusted.

Unlike the tactile sensors of previous studies replicating simplified CBE conditions (Egorov and Sarvazyan, 2008; Gwilliam et al., 2010; Mojra et al., 2012), the proposed sensor can change its effective stiffness by altering its internal pressure. Figure 14 shows the average root-mean-squared-error of the learning model as a function of internal pressure when tasked with classifying lumps by depth and diameter. The internal pressure, and therefore the effective stiffness of the tactile membrane, influences the sensitivity of the sensor. The minima present in the average error curves can be understood by plotting curves for subsets of the lump trials. Shallower lumps are more accurately identified with lower internal pressure, whereas accuracy for deeper lumps continues to improve as internal pressure increases. Accuracy for both small and large diameter lumps improves with internal pressure up until 150mbar, after which the accuracy for larger lumps decreases.

## 4 Discussion and Future Work

Our proposed tactile sensor outputs a Voronoi representation of the contacted stimuli, which has a wide range of downstream applications. In this work, we used features extracted from the Voronoi diagram as inputs to a statistical machine learning model, trained for the task of lump detection. Experimental results demonstrated the sensor’s ability to successfully identify anomalous lumps located both on a flat surface and embedded within a silicone matrix. These results highlight the potential for this method of tactile sensing to be applied in medical diagnosis and surgery, where versatile soft sensing and actuation is required and use of harsher (e.g., x-ray) technology is not permitted. More widely, there is scope for application in other robotic tasks involving dexterous manipulation of delicate objects.

The sensor’s ability to resolve surface and embedded lumps was investigated on lumps ranging from 1 to 5mm in diameter and 1 to 5mm depth in a soft silicone matrix. The sensor was capable of both detecting and evaluating the size and depth of lumps across the full range of lumps tested. The ability of the sensor to detect surface lumps was reasonably uniform across the range of lump sizes, which suggests that satisfactory performance could also be obtained outside of the tested range. Future work will investigate the upper and lower bound of the sensor’s ability to resolve surface detail. Lumps embedded within a silicone matrix proved more difficult for the sensor to accurately identify. Lumps located closer to the surface and with a smaller diameter were easier for the sensor to distinguish. With a thinner layer of silicone between the firm lump and the tactile membrane, lumps embedded nearer to the surface caused a greater change in the detectable deformation of the membrane. When palpating smaller lumps, the change in tactile membrane deformation was more localized, and therefore easier to distinguish from the large scale deformation due to the soft matrix. The observed reduction in detection performance for larger diameter lumps is assumed to be due to constraining the experiments to central indentation. If a more complex surface scanning methodology were adopted, the edges of larger lumps could be detected as the surface is traversed. The accuracy of identifying both surface and embedded lumps increased with the normal indentation force exerted into the test slab. This effect was more pronounced in the variable depth trials than the variable diameter trials. This matches the assumption that a greater force would be required to compress the soft silicone above a deeper lump before its geometry is clearly identifiable.

In our experiments, pressure data were synchronized with image data and used to identify the frame corresponding to the point of maximum membrane indentation. However, this is far from the extent of the utility of the pneumatic subsystem within the sensor. Our proposed sensor can actuate its contact surface by varying its internal pressure, thereby changing the effective stiffness. Experiments were carried out with the internal pressure ranging from 0 to 200mbar. At 200mbar, the volume of the tactile membrane increased by 60%. This potential for actuation of the contact surface may facilitate the use of other medical diagnosis techniques complementary to an automated CBE, such as nipple aspirate fluid collection (Shaheed et al., 2017). When detecting surface lumps, performance at different membrane stiffnesses was a function of indentation force. With light indentation force, a low-stiffness membrane, able to readily deform to the lump, performed significantly better than a high-stiffness membrane. This disparity in sensing performance across the stiffness range decreased as the indentation force was increased. At the highest indentation forces, where a certain internal pressure is required to resist larger scale deformation and remain conformed to the shape of the lump, the lowest membrane stiffnesses performed worse. The membrane stiffness also affected the sensor’s ability to detect embedded lumps. Noteworthy is the presence of minima in the prediction error against internal pressure curves for both the depth and diameter sensing tasks. This suggests that depending on the sensing task and the nature of the stimuli to be detected, different membrane effective stiffnesses can be considered optimal. With knowledge of the stimuli of interest, tuning the stiffness of tactile sensors could lead to improved performance or in cases where the stimuli to be detected is unknown, a methodology sweeping across effective stiffnesses while repeating palpations may be favourable. In future work, we will carry out further experimental investigation and look to characterize how the effective stiffness of the tactile membrane impacts sensing performance across a much larger and more varied sample size of trials.

The primary motivating factor behind averaging features axisymmetrically and constraining experiments to central indentation was the limited experimental sample size of approximately 4,000 observations for each experiment, which was small for a complex machine learning task. It also informed our choice of simple linear regression and Gaussian classifier models, which are suitable for identifying broad trends in small data sets while limiting overfitting. By increasing the size of the data set and training a more complex model, capable of learning the many non-linear behaviors inherent in soft body interactions, the performance of the sensor would likely improve, with no changes to the hardware. With more data and a more complex model, the number of training features could also be increased to remove the axisymmetric assumption. The additional spatial dimension would reveal information about the position and orientation of stimuli, and enable the modeling of more complex tactile interactions. Deep convolutional neural networks have shown promising performance in combination with raw optical tactile data (Lepora et al., 2018) and may provide a research avenue for improving sensor versatility in future work.

To the best our knowledge, our prototype sensor’s novel design combines optical marker-based tactile sensing with variable pressure in a significantly smaller footprint than any other device to achieve this in the literature. The sensor uses low cost manufacturing techniques and allows for the tactile membrane to be removed and replaced as a modular component. This addresses common concerns surrounding tactile sensors of high cost, low modularity, fragility and incompatibility with medical applications that require disposable contact surfaces (Tiwana et al., 2012; Zou et al., 2017). As the tactile membrane is modular and machine learning algorithms can be trained to new stimuli, future work will retain the proposed sensor design while investigating how changes to the thickness and material properties of the tactile membrane influence performance across different ranges of force applicable for clinical use.

Where previous studies have used the combination of tactile sensing and variable pressure in reactive grasping, shape identification and stiffness evaluation tasks, we utilize our control over internal cavity pressure to investigate the potential of such a sensor against a simplified mock-up inspired by the sensing requirements of CBEs. The mock-up encapsulates the challenge of CBEs of detecting firm lumps embedded within a softer matrix and as such provides a starting point from which future work can expand on exploring the role of variable compliance in CBE applications. The consistency of breast tissue varies widely (Bickley et al., 2013), so while no single breast model can be perfectly representative of all women, further experiments against a range of realistic stimuli will enable comparisons to be drawn between the sensor’s performance, human sensing ability and the results of previous tactile sensing studies (Egorov and Sarvazyan, 2008; Gwilliam et al., 2010; Mojra et al., 2012). Future work will test performance using medical breast phantoms involving non-flat surfaces, and followed by clinical trials. Testing will also be expanded to investigating stimuli other than lumps as well as exploring its potential in actively actuated palpation.

## Data Availability

All underlying data to support the conclusions are provided within this paper.
